# Fabrication of silane-modified magnetic nano sorbent for enhanced ultrasonic wave driven removal of methylene blue from aqueous media: Isotherms, kinetics, and thermodynamic mechanistic studies

**DOI:** 10.3906/kim-2007-66

**Published:** 2021-02-17

**Authors:** Abdullah ., Esra ALVEROĞLU DURUCU, Aamna BALOUCH, Ali Muhammad MAHAR

**Affiliations:** 1 National Centre of Excellence in Analytical Chemistry, University of Sindh Jamshoro Pakistan; 2 İstanbul Technical University, Faculty of Science and Letters, Department of Physics Engineering, İstanbul Turkey

**Keywords:** Ultrasonic wave, Fe_3_O_4_@SiO_2_, methylene blue, tetraethyl orthosilicate

## Abstract

In this study, we report a simple and economic one-pot synthesis of magnetite (Fe_3_O_4_) nanostructure and its modification with tetraethyl orthosilicate by coprecipitation method. The synthesized (Fe_3_O_4_@SiO_2_) nano sorbent was applied for enhanced adsorptive removal of methylene blue by ultrasonic wave driven batch experiments. After successful synthesis, the nanostructure was characterized for their physical structure by FT-IR, VSM, TEM, and XRD. For the maximum adsorptive performance of nano sorbent, various parameters were optimized, such as dose, pH, time, concentration, and temperature. The adsorption mechanism was best fitted by Langmuir isotherm with a maximum capacity of 148.69 mg/g, while kinetics best fitted by pseudo-second-order kinetic. The synthesized nano sorbent was successfully applied for enhanced adsorptive removal of toxic methylene blue from aqueous media. The proposed method is promising and effective in terms of simplicity, cost operation, green energy consumption, reproducible, excellent reusability, and magnetically separability with fast kinetic.

## 1. Introduction:

Different types of organic and inorganic dyes are designed and used in textile, plastics, printing, and paper industries as colorants [u1d2f]. The effluent discharge without any proper treatment can cause health problems [u1d32]. In the last few decades, the removal of dye from textile effluents has been a challenge. Therefore, there is a need to develop effective methods for dyes removal from wastewater [7].

Various chemical, biological, and physical methods such as reverse osmosis, precipitation, electrochemical treatment, biodegradation, and adsorption have been developed to remove these dyes, but these methods are costly [8]. Among this treatment, the adsorption process is versatile and superior to remove toxic dyes from wastewater because of its simplicity, ease of operation, low cost, high adsorption capacity, reliability, and less energy consumption [9,10]. In the last decade, various nanomaterials have been designed and employed as nano sorbents for wastewater treatment [u1d34]. Metal-organic frameworks were found effective due to high surface area with active saturated and unsaturated metal sites. But these sorbents has some limitation due to not being easy to be recovered from aqueous media [u1d35].

But, Fe_3_O_4_ NPs are easily oxidized and leached in solution in acidic media, which aggregate and lead to anisotropic kind of dipolar attraction [18]. Silica coating is considered more reliable in comparison with another surface coating due to high stability and abundance surface hydroxyl groups, which provide more chances for further attachment of another functional group. Currently, Sobia et al. used silica caped magnetite NPs for methylene blue dye from aqueous media with a maximum adsorption capacity of 123 mg/g at pH = 10, contact time 60 = min, and adsorbent dose = 30 mg [2]. Recently, ultrasonic energy got more attention due to its being safe. Ultrasonic energy enhances the chances of the interaction between reacting species via good dispersion and reduces batch experimental time by improving mass transfer.

The ultrasonic waves lead to an alternating adiabatic compression and rarefaction cycle of the liquid media, which decrease the liquid film thickness attached to the solid phase and mass transfer resistances [21-23].

In this work, we report the synthesis of silane-modified magnetic nanoparticles by a simple one-pot liquid phase coprecipitation method. By taking advantage of ultrasonic energy, we applied the silane-modified magnetic nano sorbent for enhanced removal of methylene blue dye from the aqueous system. Ultrasonic waves agitation dispersed the nano sorbent completely in solution, which increases the chances of sorbent interaction with the analyte more than usual shaking, which improves the sorption efficiency. Furthermore, various parameters such as adsorbent dose, time, pH, concentration, and temperature were optimized.

## 2. Experimental section

### 2.1 Chemical reagents and glassware

All chemicals used during this study were analytical grade. Ferrous chloride tetrahydrate salts (FeCl_2_.4H_2_O), tetraethyl orthosilicate (TEOS), and ferric chloride hexahydrate (FeCl_3_.6H_2_O) were obtained from Sigma-Aldrich (Sigma-Aldrich Corp., St. Louis, MO, USA). Ethanol was obtained from Dae-Jung (Korea). Sodium hydroxide (NaOH), hydrochloric acid 37% (HCl), nitric acid (HNO_3_), ammonium hydroxide (NH_4_OH) were purchased from Merck (Merck& Co. Inc., Kenilworth, NJ, USA). All the glassware was soaked in 10% HNO_3_ solution overnight to remove possible contamination, and was finally washed with distilled water and dried at 110 °C in an oven before use.

### 2.2 Synthesis of magnetite nanoparticles

Magnetite (Fe_3_O_4_) nanoparticles were prepared successfully via ultrasonic-assisted coprecipitation protocol. Precursor salts Fe^3+^ (0.06 M), Fe^2+^ (0.03 M) solution was sonicated for 30 min in three-neck volumetric flask for completed dissolution at 80 °C. After that, 20 mL of ammonium hydroxide was added, and the color changed from orange to black. The reaction lasted for 30 min with continuous mechanical stirring and sonication until complete precipitation. Furthermore, the black precipitates were removed by using an external magnet. The magnetite nanoparticles were washed 2-3 times with milli-Q water and dried later.

### 2.3 Modification of Fe_3_O_4_ with tetra ethoxy orthosilicate (TEOS)

Magnetite Fe_3_O_4_ nanoparticles were modified with silane group by an ultrasonic-assisted protocol for this purpose; 0.5 g of dried NPs was dispersed in 80 mL ethanol. After that, 20 mL NH_4_OH solution was added dropwise and solution was sonicated for 20 min. Later, 2.0 mL of tetra ethoxy orthosilicate TEOS was added. The solution was further sonicated for 90 min by keeping temperature at 70 °C. After completion of the reaction, the particles were washed with mixed water/ethanol solution three times, and dried at 90 °C for 1 h in oven.

### 2.4 Instrumentation

UV-visible spectrophotometer was used throughout the whole experiment (Biochrom Libra S22). A Metrohm 781 pH meter was used. Milli-Q water (ultrapure) was used (Elga Co. USA) throughout the experimental work. Mechanical stirrer, electronic balance, ultrasonicator, and heating instruments were used. FTIR spectrophotometer (4000–400 cm−1) was used for functional group analysis with a deuterated triglycine sulfate detector (Thermo Nicolet 5700). A transmission electron microscope with resolution 1.4 to 4Å was used for surface morphology investigation (Model Philips CM 12 TEM). X-ray diffractometer (XRD, Bruker D8), was used for phase identification and the crystalline nature of materials. For magnetic properties assessment, vibrating sample magnetometer (VSM) with an external magnetic field of ±10 kOe was used.

### 2.5 Sample collection and pretreatment

Real water samples were collected from phuleli canal Sindh Hyderabad, Pakistan from different locations where receive wastewater from industrial zone. Five different samples were collected in cleaned plastic bottle. The suspended particles were filtered, and the sample was put into plastic bottles, labeled, and stored in freezer at about 4°C before analysis.

### 2.6 Ultrasonic wave-driven batch experimentations

The new ultrasonic wave batch route was used for adsorption of methylene blue (MB) from aqueous media. 0.015 g of Fe_3_O_4_@SiO_2_ nano sorbent was added to 20 mL beaker having a certain amount of MB under optimized conditions. 0.1 M NaOH/HCl was used to maintain the pH. The solution was ultrasonicated for a certain time, then the particles were separated by an external magnet, and the concentration of no adsorbed MB was analyzed by UV-visible spectrophotometer at working wavelength (*λmax*= 664 nm).

The following equations were employed for percent adsorption (E) and adsorption capacity Qe (mg/g) calculation.

(1)E=Ci-CfCix100

(2)Qe=(Ci-Cf)VW

where Ci and Cf (mg/L) are the initial and final concentration of (MB) respectively, V (mL) is the volume, W (g) is the weight of the sorbent.

## 3. Results and Ddiscussion:

### 3.1. FT-IR analysis

**Figure 1:**
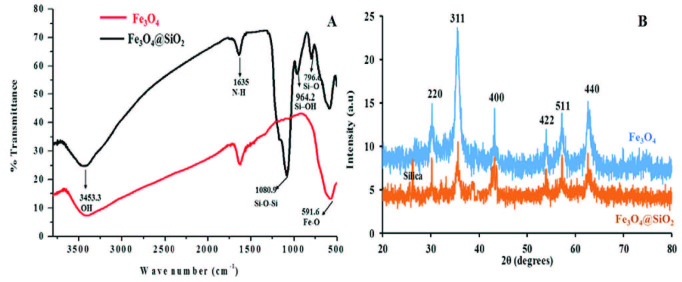
(A) FT-IR spectra of Fe3O4(Red) and Fe3O4@SiO2 (Black) nanoparticles (B) XRD patterns of Fe3O4 and Fe3O4@SiO2 nanoparticles.

The surface functionality of synthesized magnetite and silane-modified NPs was analyzed by using the FTIR spectrometer and results are shown in Figure 1(A). The broadband at 3453.3 cm^-1^ attributed to O-H stretching vibration is clearly shown in overlay spectra of both bare and silane-modified magnetite NPs. The characteristic bands in both spectra at 591.6 cm^-1^ were assigned to Fe-O antisymmetric stretching vibration [24]. The peaks at 1635 cm^-1^ attributed to N-H stretching vibration in both spectra. The sharp peaks with two shoulder peaks could be seen in the black spectrum at 1080.9, 964.2, and 796.4 cm^-1^, which correspond to Fe–O–Si, Si–O, and Si-OH stretching vibration respectively [24,25]. The presence of these peaks confirmed the successful silane modification of magnetite nanoparticles.

### 3.2. XRD analysis

The crystallinity pattern of synthesized magnetite nanoparticles before and after silane medication was examined by X-ray powder diffraction. The prominent peaks at planes (220), (311), (400), (422), (511), (440) confirmed magnetite NPs. The diffraction peaks appearance at certain points suggest that the sample is face-centered, and these results were matched with (JCPDS No. 9005837), without any noticeable trace of impurities [u1e13]. After silane modification, the same diffraction peaks were observed with slightly reduced intensities due to the silane layer on the surface of magnetite NPs, and the results are shown in Figure 1 (B).

### 3.3. TEM analysis

**Figure 2:**
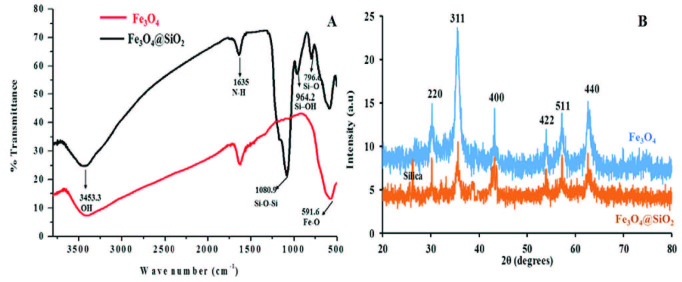
(A) TEM images of Fe_3_O_4_.(B)Fe_3_O_4_@SiO_2_ nanoparticles. (C)M~H curves of bare Fe_3_O_4_ and Fe_3_O_4_@SiO_2_ nanoparticles at room temperature.

To assess the surface morphology of synthesized nanoparticles, transmission electron microscopy was carried, and the results are shown in Figure 2A, 2B. It could be seen clearly that magnetite is highly aggregated due to its magnetic property, and, as a result, it forms big clusters making them unsuitable for the desired result and specific analytical practicality. After silane modification, the particles aggregation reduced, and the particles became spherical, well isolated, and facet in shape [24,25].

### 3.4. VSM study

VSM study was carried out to check the magnetic properties of Fe_3_O_4_ NPs before and after silane modification at room temperature using an external magnetic field of ±10 kOe; the results are shown in Figure 2(C). The saturation magnetization value of magnetite and silane-modified nanoparticles was found to be 60 and 44 emu/g, respectively. The saturation magnetization value of magnetite nanoparticles decreased after silane-modified nanoparticles, which may be due to the dead layer on the nanoparticles [25].

### 3.5. Optimization

#### 3.5.1. pH value optimization

**Figure 3:**
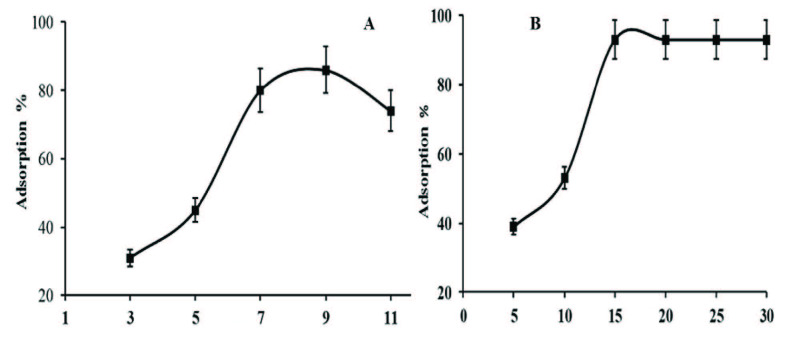
(A) Effect of pH on the adsorption of MB dye. (B) effect of dose on the adsorption of MB dye.

The pH value of the solution during the adsorption process depends on the surface charges on the analyte and adsorbent, which can be affected by changing the pH of the solution. The pH study was carried out in the range of 3 to 11 by adjusting the pH value of the solution using equimolar acid and base (0.1M HCl and NaOH). The acid and base were added in solution dropwise. It could be seen in Figure 3(A), that increasing pH value from 3 to 9 the adsorption % increased and then deceased to pH > 9. In an acidic medium, the adsorbent surface becomes more positive, which results in the repulsion of positive charged MB dye. The excess of H^+^ ions in the medium can compete at low pH value toward the adsorbent surface, which results from low adsorption of the analyte, while adsorption increases as the pH increases due to the more negative surface charge, which attracts positive charge MB with the strong electrostatic force of attraction enhancing the adsorption. The decrease in adsorption capacity of adsorbent at pH > 9 can be attributed to a loss in surface negativity of adsorbent due to the hydrolysis of MB in the excess of OH^-1^ ions in the medium, and further study was carried out at pH = 9.

#### 3.5.2. Dose optimization

Dose optimization plays a key role in describing the adsorbent loading capacity for a specific sorbate concentration. This study was carried out at a fixed concentration of sorbate by changing a sorbent dose in the range of 5-30 mg as shown in Figure 3(B). It is evident from the graph that initially the percent adsorption of MB increases with increasing the adsorbent dose but using high dose of adsorbent made the adsorption become constant, which results decrease in adsorption mass per unit of adsorbent. This could be due to the fact that the decrease in adsorption per unit time is the saturation of active sites, while increase in dose of material the percent adsorption became constant. Therefore, 0.015g of sorbent was applied for further study.

### 3.6. Isotherms study

The isotherm study was carried out to investigate the relationship between the adsorbate concentration and its accumulation pattern on the adsorbent surface at a constant temperature. 0.015 g of adsorbent was used under optimized conditions while keeping the concentration range in 5-30 mg/L. The Langmuir and Freundlich isotherm models were employed to evaluate the experimental sorption data by using the equation given below.

(3)Ce/qe=1/(qmaxb)+Ce/qemax

(4)logqe = log kf + (1/n) log Ce

**Figure 4:**
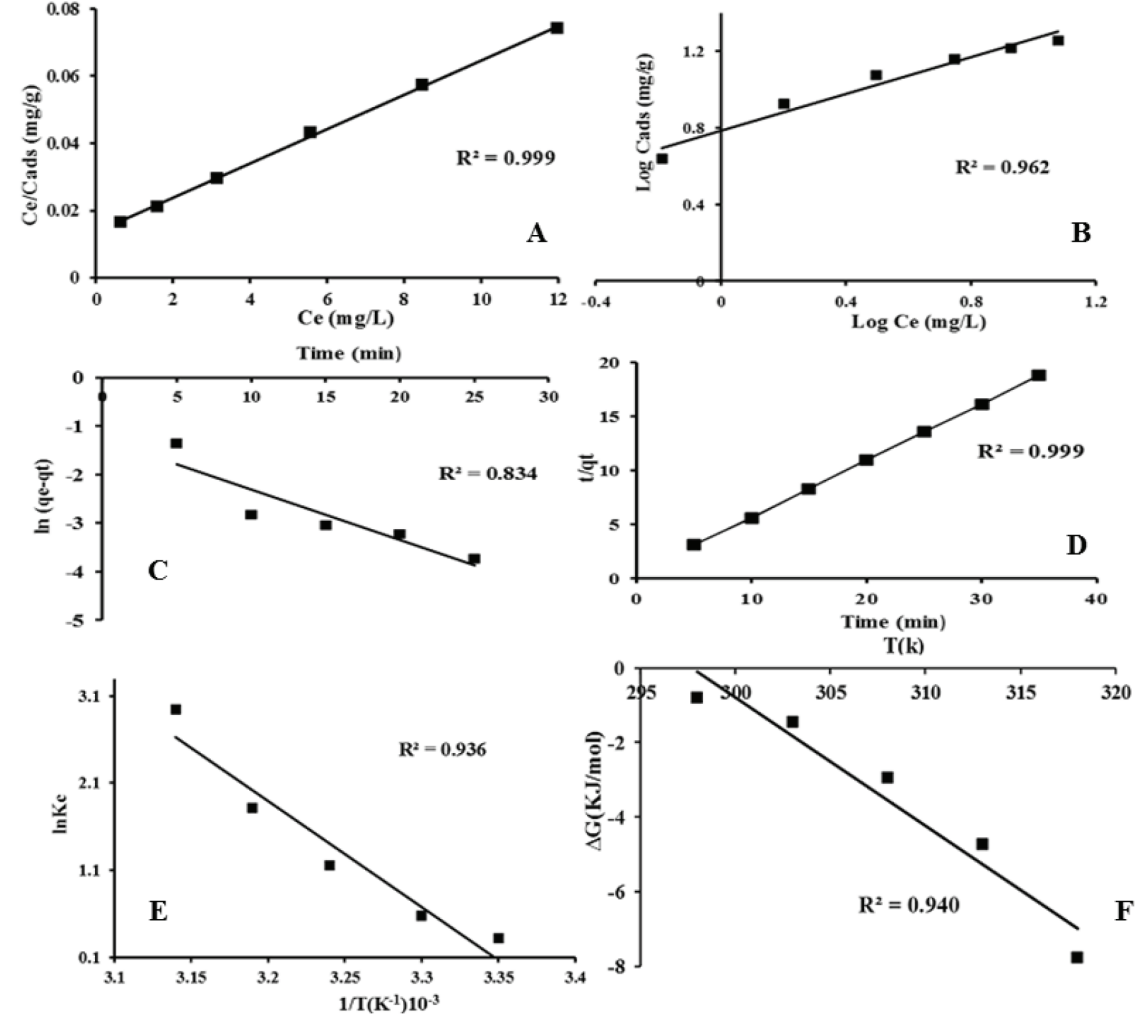
(A). Langmuir, (B). Freundlich isotherm models for Mb dye adsorption on Fe_3_O_4_@SiO_2_, (C). Pseudo first order, (D). Pseudo second-order kinetic model, (E). Van’t Hoff plot, log kc versus 1/T and (F) Temperature effect on ΔG°.

The straight line was observed by plotting Ce versus Ce/C_ads_ values as shown in Figure 4(A). From the slope and intercept of straight-line, Langmuir parameters such as maximum adsorption capacity (Q) and sorption enthalpy (b) were evaluated as shown in Table 1. Separation factor (R_L_) was found in the range of (0.081-0.346). The log C_e_ and log Cads values were plotted which becomes linear, and Freundlich parameters were assessed from this linear plot as shown in Figure 4(B). It could be seen clearly from the results that R^2^ value for Langmuir and Freundlich models were (0.999) and (0.962), respectively, which indicates that adsorption data is best described by the Langmuir monolayer model with homogenous surface, and the adsorbate is adsorbed at a well-defined active site.

**Table 1:** Langmuir and Freundlich isotherm constants for the adsorption of MB dye.

Langmuir				Freundlich			
q_0_ (mgg^-1^)	b(Lmg^-1^)	RL	R^2^	n	1/n	Kf	R^2^
148.69	0.377	0.081-0.346	0.999	2.09	0.477	6.08	0.962

### 3.7. Adsorption kinetic

The kinetic study was carried out to evaluate the binding efficiency of the sorbent with respect to time. For this purpose, different solutions of the constant concentration, 5µg/mL (10 mL) of methylene blue were prepared. Then, 0.015 g of adsorbent material were added in each solution while other experimental parameters were kept constant. The binding efficiency variation of adsorbent material was evaluated with respect to time interval of 5, 10, 15, 20, 25, 30, and 35 min . For this purpose, two kinetic models were used such as pseudo-first order and pseudo-second order. From the linear rate equation of both the model’s correlation values and rate, constants were calculated by the equations given below.

(5)log(qe-qt)=log qe -k1t2.303

(6)tq=1k2q2e+tqe

It was concluded from the results that the qe and R^2^ values of the pseudo-second-order kinetic model are higher as compared to a pseudo-first-order kinetic model as data show in Table 2 and Figure 4C. The slope and intercept of linear plots rate constants (K_1_&K_2_) are shown in Figure 4(D), and their values are given in Table 2. The results show that the experimental data is best described by pseudo-second-order kinetics, and the reaction depends upon substrate and analyte concentration.

**Table 2:** Various kinetic parameters of pseudo first order kinetics and pseudo second order kinetics for the adsorption of MB dye.

Pseudo first order			Pseudo second order			
K_1_(min^-1^)	qe(mg/g)	R^2^	K_2_ (mg^-1^ min^-1^)		qe(mg/g)	R^2^
0.238	0.245	0.834	0.655		1.903	0.999

### 3.8. Thermodynamics study

To assess the temperature effect on the adsorption capacity in the range of 298-318K, the thermodynamic study was observed. 0.015 g of sorbent was added in 10 mL MB solution having 5µg/mL concentration and ultrasonicated under optimized condition. Different thermodynamic parameters such as a change in enthalpy (ΔH^0^), free energy (ΔG^0^), and entropy(ΔS^0^) were evaluated using the following equations.

(7)ΔG0=-RTlnk

(8)ΔS0=ΔH0-ΔG0T

lnk=ΔH0RT+ΔS0R

The numerical values of ΔH^0^ and ΔS^0^ have been calculated from the slope and intercept of the plot as shown in Figure 4(E), and their values are given in Table (3). It could be seen in Figure 4(F) that the negative value of DG^0^ increases with increasing temperature, which describes that at higher temperature, sorption is more favorable and spontaneous. The positive values of ΔH^0^, ΔS^0^ describe that the adsorption process is endothermic, and a decline in the randomness at the solution/solid interface occurred.

**Table 3:** Thermodynamic parameters of MB adsorption at different temperatures.

T(K)	ΔG^0^(kJ/mol)	ΔH0 (kJ/mol)	ΔS^0^ (kJ/mol k)	R^2^
298	-0.799	101.23	0.339	0.936
303	-.448			
308	-2.950			
313	-4.721			
318	-7.780			

### 3.9. Repeatability study

**Figure 5:**
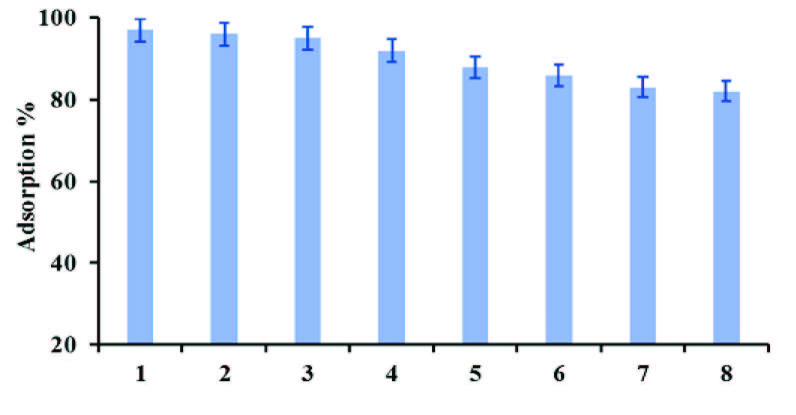
Repeated study of Fe_3_O_4_@SiO_2_ nano sorbent.

The reusability study of developed nano sorbent (Fe_3_O_4_@SiO_2_) was evaluated by the adsorption/desorption batch experimentation. In typical experimental protocol, different molar concentrations of HCL, such as 0.07, 0.08, 0.09. 0.1, and 0.2 for desorption to recover the adsorbed analyte during reusability study were applied. The maximum recovery about 95% was achieved at using 0.1 and 0.2M HCl. Therefore, 0.1 M HCl was used for all desorption experiments; the final concentration of MB was determined by UV-Visible spectrophotometer. Afterwards, an excellent recovery with an insignificant decrease by less than 10% in their binding capability was attained by reusing the same sorbent seven times as displayed in Figure (5).

### 3.10. Interfering effect

The selectivity and adsorptive performance of silane-modified magnetic nonabsorbent was carried out using different analyte/interferent concentration ratios such as (1:1, 1:10) mg/L under optimized condition. During this study, all the interferent compounds such as thymol blue, methyl red, rhodamine b, and methyl orange were spiked in the same solution and the effect of different interferent dyes on the percent adsorptive removal of methylene blue were studied; the results are presented in the table (4). It was assessed from results that methylene blue could be removed by using silane-modified magnetic nonabsorbent efficiently in the presence of other dyes due to the smaller size of methylene blue and its strong bonding with silane group on the surface of magnetic nonabsorbent.

**Table 4:** Effect of interfering dyes on the sorption of 1 mgL-1methylene blue.

Interfering dyes	Analyte/interferent ratio(mg/L)	% Recovery
Thymol blue	1:2 1:10	99 97.5
Methyl orange	1:2 1:10	98.4 96.5
Rhodamine b	1:2 1:10	98.2 94.3
Methyl red	1:2 1:10	98 95.3

### 3.11. Comparison of some previously reported studies

The analytical features and applicability of silane-modified (Fe_3_O_4_@SiO_2_) ultrasonic wave driven nano sorbent for enhanced adsorptive removal MB in the aqueous system were compared to previously reported adsorbent for MB removal as presented in table 5, [26-35]. The comparative study table shows that ultrasonic mediated silane-modified nano sorbent is effective in terms of linear range, fast kinetic, high adsorption capacity, and it is magnetically separable within 14 s from aqueous solution providing excellent reusability of the same material for seven successive cycles with a negligible decrease in their adsorption capacity by less than 10%.

**Table 5:** Comparison of different adsorbents used for MB dye removal.

Adsorbents	Dyes	pH value	T (min)	Qmax (mg/g)	Dose (mg)	Ref
PANI hydrogel	MB	6.5	180	71.20	20	[26]
Polyzwitterionic resin	MB	7	-	14.9	30	[27]
Titania-incorporated polyamide	MB	6	30	43	20	[28]
PTMP	MB	5	8	64.5	20	[29]
A pH-responsive resin	MB	3-7	30	-	20	[30]
PDA	MB	7-10	60	90.7	30	[31]
Fe_2_O_3_-ZrO_2_/BC	MB	-	-	38.10	-	[32]
Fe_3_O_4_@SiO_2_	MB	10	60	123	30	[2]
Cellulose capped Fe_3_O_4_	MB	11	-	13.54	-	[33]
γ-Fe_2_O_3_@GL	MB	7-10	90	69.63	1000	[34]
Fe-BDC MOF	MB	9	360	8.65	25	[35]
Ultrasonic wave driven Fe_3_O_4_@SiO_2_	MB	9	30	148.69	15	Current study

### 3.12. Analytical applicability to real samples

The analytical features and practicality of Fe_3_O_4_@SiO_2_ as a magnetic solid-phase sorbent for enhanced adsorptive removal of MB from the aqueous medium were studied under optimized conditions. An excellent linear concentration range (0.25-25) μg/mL with R^2^ (0.991) was achieved. The limit of detection (LOD) (3SD/m) and limit of quantification (LOQ) (10SD/m) were obtained as 0.072 and 0.24 μg/mL, respectively, where m is the slope of the standard curve and SD is the standard deviation of 10 times of blank reading. The validation of developed nano sorbent to real water samples was carried out by spiking standard addition. We applied the developed method to 5 different real water samples collected from 5 different locations of phuleli canal Sindh Hyderabad in order to check the analytical practicality of synthesized nano sorbent. The phuleli canal receives wastewater from industrial zone and highly contaminated. Three replicates of each samples were analyzed using nano adsorbent. Many recovery batch experimentations were carried by spiking 2 μg/mL of MB in real samples by standard addition, and reasonable recoveries from 96% to 98% of MB in the real spiked samples were attained, which showed that the developed Nano sorbent is a real magnetically separable candidate for enhanced preconcentration of MB. The results are given in Table 6.

**Table 6:** Spiked recovery test of MB dye in a real water sample (n = 3).

Sample	S1	S2	S3	S4	S5
Without addition	0.0	0.0	0.0	0.0	0.0
MB added (μg/mL)	2.0	2.0	2.0	2.0	2.0
MB found (μg/mL)	1.97±0.8	1.92±0.7	1.94±0.51	1.96±0.28	1.98±0.71
% Recovery	98.5	96	97	98	99

## 4. Conclusion

In this study, we report for the synthesis of Fe_3_O_4_@SiO_2_ nano sorbent by a simple and economic coprecipitation method and applied for enhanced adsorptive removal of MB from aqueous medium by novel ultrasonic wave-driven batch experiment. Ultrasonic energy application during batch adsorption experiments dispersed the nano sorbent completely in solution, which increases the chances of sorbent interaction with the analyte compared to usual shaking. The proposed method is best in terms of operative cost, simplicity, green energy consumption, reproducibility, excellent reusability, and magnetically separability with fast kinetics. During this study, the various parameter was optimization for maximum adsorption performance i.e. contact time, Ph value, concentration, temperature, and sorbent dose. The synthesized nano sorbent was successfully applied to real water samples, and the results show that it is an excellent magnetically separable, fast kinetics candidate for enhanced adsorptive removal of methylene from aqueous media.
